# The *Caenorhabditis elegans* ARIP-4 DNA helicase couples mitochondrial surveillance to immune, detoxification, and antiviral pathways

**DOI:** 10.1073/pnas.2215966119

**Published:** 2022-11-29

**Authors:** Kai Mao, Peter Breen, Gary Ruvkun

**Affiliations:** ^a^Department of Molecular Biology, Massachusetts General Hospital, Boston, MA 02114; ^b^Department of Genetics, Harvard Medical School, Boston, MA 02115

**Keywords:** mitochondria, RNAi, detoxification, healthspan

## Abstract

Surveillance of *Caenorhabditis elegans* mitochondrial status is coupled to defense responses such as drug detoxification, immunity, antiviral RNA interference (RNAi), and regulation of life span. A cytochrome p540 detoxification gene, *cyp-14A4*, is specifically activated by mitochondrial dysfunction. The nuclear hormone receptor NHR-45 and the transcriptional Mediator component MDT-15/MED15 are required for the transcriptional activation of *cyp-14A4* by mitochondrial mutations, gene inactivations, or toxins. A genetic screen for mutations that fail to activate this cytochrome p450 gene upon drug or mutation-induced mitochondrial dysfunction identified a DNA helicase ARIP-4 that functions in concert with the NHR-45 transcriptional regulatory cascade. In response to mitochondrial dysfunction, ARIP-4 and NHR-45 protein interaction is enhanced, and they relocalize from the nuclear periphery to the interior of intestinal nuclei. NHR-45/ARIP-4 also regulates the transcriptional activation of the *eol-1* gene that encodes a decapping enzyme required for enhanced RNAi and transgene silencing of mitochondrial mutants. In the absence of *arip-4*, animals were more susceptible to the mitochondrial inhibitor antimycin. Thus, ARIP-4 serves as a transcriptional coactivator of NHR-45 to promote this defense response. A null mutation in *arip-4* extends the life span and health span of both wild type and a mitochondrial mutant, suggesting that the activation of detoxification pathways is deleterious to health when the mitochondrial dysfunction is caused by mutation that cannot be cytochrome p450-detoxified. Thus, *arip-4* acts in a pathway that couples mitochondrial surveillance to the activation of downstream immunity, detoxification, and RNAi responses.

## Significance

Eukaryotes surveil and respond to mitochondrial dysfunction caused by toxins or mutations, triggering antiviral and immune responses as well as mitochondrial repair. A mutation in the *Caenorhabditis elegans *mitochondrial chaperone *hsp-6 *activates detoxification and RNA interference defense pathways. Mitochondrial mutations increase *C. elegans *longevity, and mutations that disable RNA interference suppress this longevity increase. Mitochondria also serve as a signaling hub for mammalian immune responses to viruses. The conserved DNA helicase gene *arip-4 *is required for the increase in antiviral RNAi caused by mitochondrial dysfunction. But an *arip-4 *mutation extends the life span of the *hsp-6 *mitochondrial mutant, suggesting that the futile activation of detoxification pathways is deleterious to health when the mitochondrial dysfunction is caused by mutations.

Human aging is accompanied by diverse pathologies, including cancer, diabetes, neurodegeneration, and cardiovascular diseases, suggesting a complex and progressive decay of multiple pathways (1). A decline in cellular protection, homeostasis, and regeneration contributes to senescence (2). Despite this complexity, genetic analyses have revealed single-gene mutations that can extend life span; such mutations may trigger life span–prolonging genetic programs that are normally triggered by nutritional or pathogen cues. One of the largest class of mutations that increase longevity in the nematode *Caenorhabditis elegans* are mutations that reduce mitochondrial function (3, 4).

The mitochondrion has its origin from an ancient endosymbiotic association with an alpha-proteobacteria, and most of the few thousand bacterial genes of the original endosymbiont migrated to the nucleus where these genes are regulated no differently from other nuclear genes; just a few dozen genes remain in the mitochondrial genomes of eukaryotes. But the proteins encoded by the nuclear genes that encode mitochondrial proteins retain many of their functions for metabolism and electron transport, for example, and are localized to the mitochondria after translation on the cytoplasmic and secretory ribosomes. More than 1,000 nuclear-encoded proteins with a deep bacterial ancestry reassemble the mitochondrion from its nuclear diaspora bacterial ancestral genome in each eukaryotic cell (5). A large fraction of the nuclear encoded mitochondrial proteins retain homology to modern bacterial proteins. Most bacterial species and most eukaryotic mitochondria generate energy via proton pumping across the mitochondrial membranes by the many heme and iron–sulfur proteins of the electron transport chain (ETC). The free energy of this ETC-generated proton gradient drives the synthesis of adenosine triphosphate (ATP) by the F_1_F_0_-ATP synthase to power thousands of enzymatic reactions. The bacterial origin of the eukaryotic mitochondrion allows the highly evolved weapons of 4 billion years of bacterial competition between each other to be marshaled by bacterial pathogens against eukaryotes. Because iron became a limiting factor in many ecosystems after the evolution of bacterial photosynthesis, the mitochondrion is an attractive target of bacterial pathogens that may find the bacterial lineage of mitochondria familiar and vulnerable to their highly evolved antibacterial virulence weapons (6, 7).

The mitochondria also serve as a key signaling hub for immune responses to viruses. For example, the mammalian mitochondrial antiviral signaling protein (MAVS) associates with viral RNA sensor retinoic acid–inducible gene I (RIG-I) or melanoma differentiation–associated protein 5 (MDA5) to trigger the downstream nuclear factor kappa B, as well as other interferon immune signaling (8). In addition, the mitochondrion harbors its own idiosyncratic genome encoding just 13 protein-coding genes, 22 tRNA genes, and two ribosomal RNA genes. DNA or RNA released from the mitochondria may share more features with the nucleic acids of viruses than the nuclear genes, and is interpreted as “foreign,” eliciting immune responses through RIG-I or MDA5 and MAVS (9, 10).

RNA interference (RNAi) is highly conserved across most eukaryotes. It deploys 22- to 26-nt single-stranded small interfering RNAs (siRNAs) produced by Argonaute proteins and the Dicer dsRNA ribonuclease, as well as RNA-dependent RNA polymerases in a large fraction of eukaryotes but in a small fraction of animal genomes (11). These siRNAs target mRNAs for degradation or chromatin regions for epigenetic silencing across most eukaryotes (11). RNAi was first identified in *C. elegans* and plants by its antiviral activities that detect and cleave foreign nucleic acids such as viruses (12, 13). The *C. elegans* RNA helicase DRH-1 that is orthologous to mammalian RIG-I or MDA5 regulates antiviral RNAi responses to degrade invading viruses (14, 15). In mammalian cells, release of mRNAs transcribed from the mitochondrial genome into the cytosol is an immune elicitor (16); similar activation of immune response by mitochondrial RNA release has been observed in *Drosophila melanogaster* and *C. elegans* (17, 18). In *C. elegans*, mitochondrial dysfunction due to a mutation in the mitochondrial chaperone gene *hsp-6* causes an enhanced level of RNAi and transgene silencing that is a classic feature of the induction of antiviral RNAi (18, 19).

Over the past two billion years, eukaryotes have evolved signaling networks to detect mitochondrial dysfunction, caused either by toxins from pathogens or by mutations that can be misconstrued by toxin or pathogen surveillance pathways as a pathogen attack, to trigger defense of their mitochondria. In *C. elegans*, the mitochondrial unfolded protein response (UPR^mt^) is a mitochondria-to-nuclear communication channel that is activated by various types of mitochondrial dysfunction, including natural toxins such as oligomycin or mutations in mitochondrially localized nuclear-encoded proteins (20). The transcriptional outputs of UPR^mt^ include mitochondrial chaperones, proteases, detoxifying enzymes, and secreted antibacterial proteins that enable defense and recovery of mitochondrial function. Detection of *C. elegans* mitochondrial dysfunction couples to the coordinated induction of drug detoxification and immune response genes (21–23). We previously identified a nuclear hormone receptor (NHR) NHR-45 and a Mediator component MDT-15/MED15 as components of a dedicated transcriptional pathway for mitochondrial dysfunction responses (23).

Here, we report the identification of the conserved DNA helicase RAD-26/ARIP-4 in a large genetic screen for mutants that fail to activate detoxification response to mitochondrial dysfunction. *arip-4* is required for the activation of the cytochrome p450 xenobiotic detoxification gene *cyp-14A4*, normally activated by mitochondrial dysfunction. We find that the ARIP-4 DNA helicase associates with the NHR-45 to transcriptionally activate drug detoxification and immunity genes and mediate resistance to the mitochondrial inhibitor antimycin or pathogenic bacteria *Pseudomonas aeruginosa*. The NHR-45/ARIP-4 pathway also regulates the increase in antiviral RNAi response caused by mitochondrial dysfunction through induction of the decapping enzyme gene *eol-1*. A null mutation in *arip-4* extends the life span and the health span of both wild type and a mitochondrial mutant, suggesting that the activation of detoxification pathways is deleterious to health when the mitochondrial dysfunction is caused by mutation that cannot be cytochrome p450-detoxified rather than by a toxin.

## Results

### Activation of the Xenobiotic Reporter *cyp-14A4p::gfp* Requires RAD-26/ARIP-4.

Surveillance of mitochondrial integrity is essential for maintaining cellular homeostasis and is coupled to a complex signaling network that induces mitochondrial recovery, drug detoxification, and immunity. Although almost all of the known mitochondrial stress–related transcriptional responses required the UPR^mt^, certain responses, such as detoxification and immunity, needed additional factors (24). There are about 250 *C. elegans* drug detoxification genes of four major classes: cytochrome p450, uridine diphosphate (UDP) glycosylating, sulfate and glutathione S-transferase (GST) genes that add hydrophilic groups to generally hydrophobic toxins, and ABC transporters that excrete the modified toxins (25). Different suites of these detoxification genes are triggered by mitochondrial dysfunction compared with those that are induced by ribosomal dysfunction or cytoskeletal dysfunction, for example (21). The many drug detoxification genes have evolved by gene duplication and divergence and by the acquisition of promoters that are activated by distinct cellular challenges. *cyp-14A4* is one of about 77 *C. elegans* cytochrome p450 genes and is induced specifically by a variety of mitochondrial mutations, gene inactivations, or toxins but not, for example, by ribosomal dysfunction or cytoskeletal dysfunction (21, 25). A fusion of the *cyp-14A4* promoter to GFP, *cyp-14A4p::gfp,* is induced in the intestine by a variety of mitochondrial mutations (23). For example, *cyp-14A4p::gfp* is induced by *hsp-6(mg585)*, a hypomorphic mutation in the mitochondrial chaperone HSP-6/mtHSP70.

**Fig. 1.**
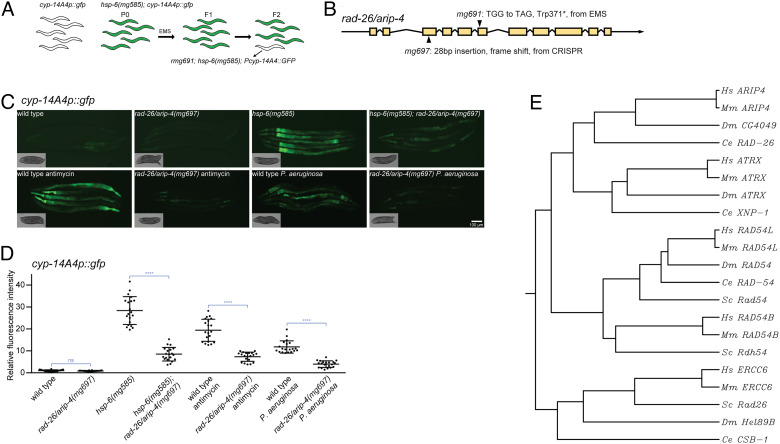
*rad-26/arip-4* gene activity is essential for the induction of *cyp-14A4p::gfp*. (*A*) The genetic screen that identified the *rad-26/arip-4(mg691)* mutant by its failure to activate *cyp-14A4p::gfp* in the *hsp-6(mg585)* mitochondrial mutant. (*B*) The *rad-26/arip-4(mg697)* mutant allele generated by CRISPR-Cas9. (*C* and *D*) The induction of *cyp-14A4p::gfp* transcriptional fusion reporter requires *rad-26/arip-4*. The *cyp-14A4p::gfp* is strongly induced by *hsp-6(mg585)*, antimycin, or *P. aeruginosa*, and the induction is abrogated by the *rad-26/arip-4(mg697)* mutant. Animals were imaged in (*C*), and the fluorescence was quantified in (*D*). Error bar: SE of the mean; **** denotes *P * < 0.0001. (*E*) Phylogenetic tree of RAD-26/ARIP-4 and members of the gene family in animals and yeast. RAD-26/ARIP-4 is conserved from worms to mammals but is more closely related to ARIP4 than other human helicases that define other branches on the tree. *Hs*, Homo sapiens; *Mm, Mus musculus*; *Dm, D. melanogaster*; *Ce, C. elegans*; and *Sc, Saccharomyces cerevisiae*.

A genome-wide ethyl methanesulfonate (EMS) mutagenesis screen for mutants that fail to induce *cyp-14A4p::gfp* in *hsp-6(mg585)* revealed an allele *mg691,* a Trp371 to Stop (TGG to TAG) nonsense mutation in *rad-26* (Fig. 1 *A* and *B*). After an EMS mutagenesis, any F2 mutant selected for any phenotype carries 100–300 distinct mutations distributed across its genome; any one of those mutations could cause the new phenotype, in this screen, failure to induce *cyp-14A4p::gfp* in the *hsp-6(mg585)* mitochondrial mutant. However, many of the EMS mutations will be located in introns or intergenic regions, and many are not predicted to cause amino acid substitutions (26). After the detection of a candidate lesion such as *rad-26*(*mg691),* it is necessary to reconstruct a similar mutation in the absence of the other EMS lesions traditionally by multiple backcrossing but recently using the CRISPR to engineer only that single lesion in an unmutagenized background.

To establish that the lesion in *rad-26* in this F2 mutant isolated after a mutagenesis screen was the causative mutation for the failure to induce *cyp-14A4p::gfp* in *hsp-6(mg585)*, CRISPR-Cas9 editing was used to generate just a single independent null allele of *rad-26(mg697)* with 28 base pairs inserted that caused a frameshift (Fig. 1*B*). The loss-of-function *rad-26(mg697)* mutant also disrupted induction of *cyp-14A4p::gfp* in the *hsp-6(mg585)* background (Fig. 1 *C* and *D*). The *rad-26* requirement for the activation of *cyp-14A4* expression was not limited to the *hsp-6(mg585)* mitochondrial mutant: The complex III natural toxin antimycin isolated from *Streptomyces* bacteria also activates *cyp-14A4p::gfp,* and this activation is dependent on *rad-26* gene activity (Fig. 1 *C* and *D*)*.* The human pathogenic bacteria *P. aeruginosa* produces chemical toxins that disrupt the mitochondrial membrane potential and virulence factors that inhibit translation (27, 28). *P. aeruginosa* also activates *cyp-14A4p::gfp* (Fig. 1 *C* and *D*), and this induction is dependent on *rad-26* (Fig. 1 *C* and *D*). Thus, *rad-26* is necessary for the coupling of mitochondrial dysfunction caused by mitochondrial mutations or toxins to the activation of the detoxification gene *cyp-14A4*.

The 1,274 amino acid proteins encoded by *rad-26* is one of 20 paralogous DNA helicases in the *C. elegans* genome. These DNA unwinding helicases hydrolyze ATP to supply free energy to break the hydrogen bonds between annealed nucleotide bases and separate double-stranded DNA into single strands (29). There are 31 DNA helicases in the human genome, classified into six groups (30). Although the *C. elegans* nomenclature RAD-26 refers to *Saccharomyces cerevisiae* Rad26 (RADiation sensitive), these two proteins are not orthologs but paralogs. They are distant from each other on the phylogenetic tree of these DNA helicases (Fig. 1*E*). *S. cerevisiae* Rad26 is responsible for transcription-coupled nucleotide excision repair (31), and its ortholog is ERCC6 in human and CSB-1 in *C. elegans* (Fig. 1*E*). The phylogenetic tree shows that *C. elegans rad-26* encodes a conserved Snf2-like DNA-dependent ATPase orthologous to human ARIP4 (androgen receptor–interacting protein) (Fig. 1*E*). Because there is essentially no literature about the genetic function of the *C. elegans rad-26* gene and because it is an ortholog of mammalian ARIP4, both based on the phylogenetic tree and based on its function with NHRs (see below), we rename this *C. elegans* gene *arip-4*.

The Snf2 family DNA helicases are involved in transcription regulation, chromatin remodeling, homologous recombination, and DNA repair (32). When participating in transcription, these DNA helicases function as coregulators and interact with transcriptional factors, including NHRs. Fitting with the *cyp-14A4* transcriptional regulatory function of *C. elegans arip-4*, and with its genetic and biochemical interaction with the *C. elegans* NHR NHR-45 (see below), human ARIP4 is a transcriptional coregulator for NHRs (33, 34). ARIP4 belongs to the Snf2 family DNA helicases and serves as a transcriptional coactivator to enhance androgen-dependent transcriptional programs (35). ARIP4 may be animal specific (Fig. 1*E*) because no ortholog exists in *S. cerevisiae* or *Arabidopsis thaliana* (32). Loss of ARIP4 in mice is embryonic lethal with increased apoptosis and decreased DNA synthesis (36).

### *C. elegans* ARIP-4 Serves as a Transcriptional Coactivator for the NHR NHR-45 that Acts in the Mitochondrial Dysfunction Detoxification Response Pathway.

The *C. elegans* NHR NHR-45 and Mediator subunit MDT-15 are required for coupling the detection of mitochondrial dysfunction to the upregulation of the *cyp-14A4* detoxification gene (23). Because the mammalian ortholog of *C. elegans* ARIP-4, ARIP4, directly interacts with NHR proteins, we hypothesized that *C. elegans* ARIP-4 may associate with NHR-45 to regulate *cyp-14A4* transcription.

**Fig. 2.**
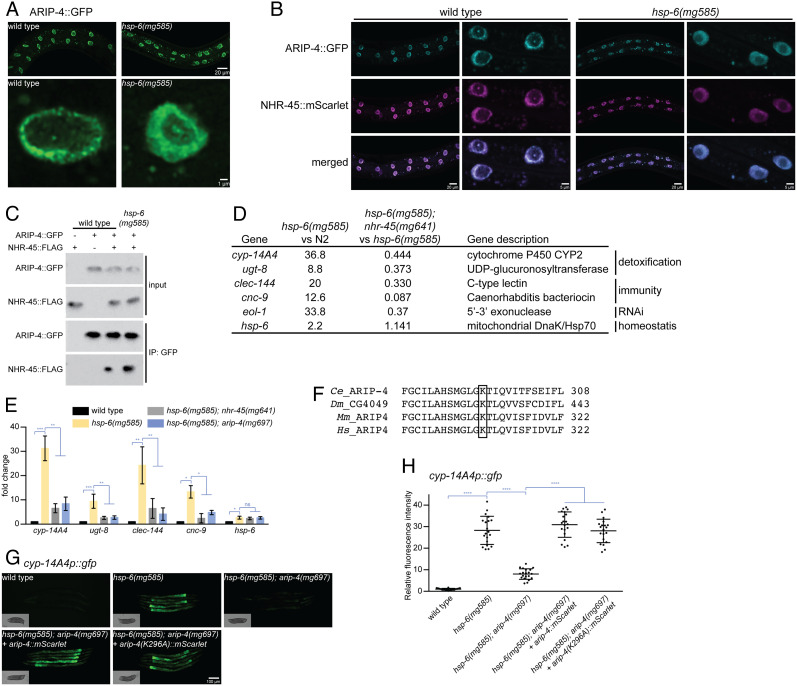
ARIP-4 protein colocalizes and associates with NHR-45 protein for transcriptional regulation. (*A*) *vha-6p::arip-4::gfp* fusion protein localizes in the nucleus with different patterns of subnuclear localization in wild type or the *hsp-6(mg585)* mitochondrial mutant. (*B*) *vha-6p::arip-4::gfp* fusion protein colocalizes with *vha-6p::nhr-45::mscarlet* fusion protein in wild type or the *hsp-6(mg585)* mitochondrial mutant. (*C*) In vivo GFP pull-down to be followed by FLAG detection of the interaction between ARIP-4::GFP and NHR-45::FLAG. (*D*) Genes up-regulated by *hsp-6(mg585)* mutant and suppressed by *nhr-45(mg641)* mutant based on mRNA-seq. (*E*) Detoxification and immunity genes but not mitochondrial homeostasis in the *hsp-6(mg585)* mutant are dependent on *nhr-45* and *arip-4*. RT-qPCR assays showed that the mRNA level of *cyp-14A4*, *ugt-8*, *clec-144*, *cnc-9*, and *hsp-6* was induced by *hsp-6(mg585)* mutant, and the induction of the first four genes requires *nhr-45* and *arip-4*. * denotes *P* < 0.05, ** denotes *P* < 0.01, *** denotes *P* < 0.001, and ns denotes *P*>0.05. (*F*) Multiple sequence alignments showing the conserved lysine within the ATP-binding site of ARIP-4. (*G* and *H*) Rescue of *arip-4 (mg697)* by wild type or the K296A mutant of ARIP-4. Pictures were taken in (*G*), and the fluorescence was quantified in (*H*). **** denotes *P* < 0.0001.

We first tested whether *C. elegans* ARIP-4 is localized to the nucleus. In mammals, the majority of detoxifying cytochrome p450 are expressed in the liver; in *C. elegans,* there is no liver, but the 32 intestinal cells may perform both gut and liver functions, such as detoxification (37). The gut is also where *C. elegans* would be expected to encounter bacterial pathogens that disrupt mitochondrial function and where immune responses to bacterial toxins and virulence factors would be expected. Consistent with the homology between the *C. elegans* gut and mammalian liver, *cyp-14A4p::gfp* is predominantly induced in the intestine upon mitochondrial dysfunction in all cell types (23). To observe *C. elegans* ARIP-4 protein localization, GFP was fused at the poorly conserved C terminus of ARIP-4 under the control of an intestine-specific promoter *vha-6p* in a miniMOS vector to generate a single-copy transgene *vha-6p::arip-4::gfp::tbb-2 3'UTR* fusion protein (38). The protein is localized to the nucleus in the intestine, where the *vha-6* promoter drives the expression of the ARIP-4::GFP fusion protein (Fig. 2*A*). Interestingly, although the ARIP-4::GFP fusion protein localized in the intestinal nucleus in both wild-type and *hsp-6(mg585)* animals, the intranuclear patterns were different (Fig. 2*A*). In the wild-type nucleus, the ARIP-4::GFP fusion protein localized to the nuclear periphery, but in the *hsp-6(mg585)* mitochondrial mutant, ARIP-4::GFP was localized to the nuclear interior (Fig. 2*A*).

We hypothesized that ARIP-4 relocalization to the nuclear interior may depend on the localization of an interacting transcription factor, for example, NHR-45. We tested whether the ARIP-4 and NHR-45 proteins localize to the same region of intestinal nuclei in wild-type or mitochondrial mutant animals. To be compatible with the ARIP-4::GFP fusion protein, NHR-45 was tagged with mScarlet at its C terminus using the intestine-specific *vha-6* promoter to drive expression. In the *hsp-6(mg585)* mitochondrial mutant, NHR-45::mScarlet showed a similar shift of localization away from the nuclear periphery (Fig. 2*B*). This change of intranuclear position is consistent with models of chromatin states: Closed chromatin with repressed transcription tends to reside at the nuclear periphery, and open chromatin with active transcription tends to be more intranuclear (39).

Not only do ARIP-4 and NHR-45 colocalize and coregulate *cyp-14A4*, ARIP-4 protein physically interacts with the NHR-45 protein in a similar manner to that of its human ortholog ARIP4, which binds to another NHR protein, the androgen receptor (35). To detect this interaction, NHR-45 was fused with FLAG tag at its C terminus driven by the *vha-6* promoter. Complexes from protein extracts of *C. elegans* were first immunoprecipitated using the tag on ARIP-4::GFP and tested for coimmunoprecipitation of NHR-45::FLAG. The two proteins interacted in wild-type animals, but the interaction was enhanced in the *hsp-6(mg585)* mitochondrial mutant (Fig. 2*C*).

mRNA-seq analyses of *hsp-6(mg585)* and *hsp-6(mg585); nhr-45(mg641)* mutants (23) revealed that a variety of detoxification and immunity genes are up-regulated in the *hsp-6(mg585)* mutant but are not up-regulated in the *hsp-6(mg585); nhr-45(mg641)* mutant (Fig. 2*D*), suggesting that these genes are regulated by NHR-45. In order to test whether ARIP-4 participates in the regulation of these genes, we tested two detoxification genes *cyp-14A4* and *ugt-8* and two immunity genes *clec-144* and *cnc-9*. Their expression levels were examined by quantitative reverse transcription PCR (RT-qPCR). All four genes were up-regulated 10–30 fold in the *hsp-6(mg585)* mitochondrial mutant, and the induction was decreased more than 70% in either the *nhr-45(mg641)* or the *arip-4(mg697)* mutant (Fig. 2*E*). Thus, ARIP-4 is a transcriptional coactivator for NHR-45 to promote the activation of detoxification and immune responses, and these proteins move from the nuclear periphery to intranuclear in a coordinated manner upon mitochondrial dysfunction.

Even though mitochondrial stress–induced detoxification and immune responses required UPR^mt^ signaling, loss of *nhr-45* had no effect on mitochondrial homeostasis, for example, activation of *hsp-6* gene expression (23). We tested the mRNA level of *hsp-6* by RT-qPCR, and the up-regulated expression of *hsp-6* in *hsp-6(mg585)* mutant was not affected by either *nhr-45(mg641)* or *arip-4(mg697)* (Fig. 2*E*), which indicates that ARIP-4, like its binding partner NHR-45, does not act in the *hsp-6* pathway to promote mitochondrial homeostasis.

As a DNA helicase, we expected that the ATPase activity of ARIP-4 would be essential for its function in regulating transcription. The ATPase activity of ARIP4 was disrupted with a lysine-to-alanine mutation at the amino acid 310 (K310A), the ATP-binding site (33). The ATP-binding region of *C. elegans* ARIP-4 is conserved, and the corresponding lysine in ARIP-4 and the K296A substitution was engineered (Fig. 2*F*). The function of wild-type ARIP-4 or ARIP-4(K296A) was assayed by the induction of *cyp-14A4p::gfp*. Wild-type ARIP-4 was able to rescue the *arip-4(mg697)* mutant and could mediate the induction of *cyp-14A4p::gfp* caused by *hsp-6(mg585)*; to our surprise, the ARIP-4(K296A) mutant was also able to rescue the *arip-4(mg697)* mutant and allow induction of *cyp-14A4p::gfp* (Fig. 2 *G* and *H*). Thus, intestinal expression of *arip-4* is able to rescue the failure to activate *cyp-14A4p::gfp* in the *hsp-6(mg585)*; *arip-4(mg697)* double mutant, suggesting that ARIP-4 regulation of *cyp-14A4* is autonomous to the intestine. But, surprisingly, ARIP-4 regulated detoxification and immune responses are independent of its DNA helicase activity.

### NHR-45/ARIP-4 Activates Enhanced RNAi through Activation of the RNA Exonuclease Gene *eol-1*.

**Fig. 3.**
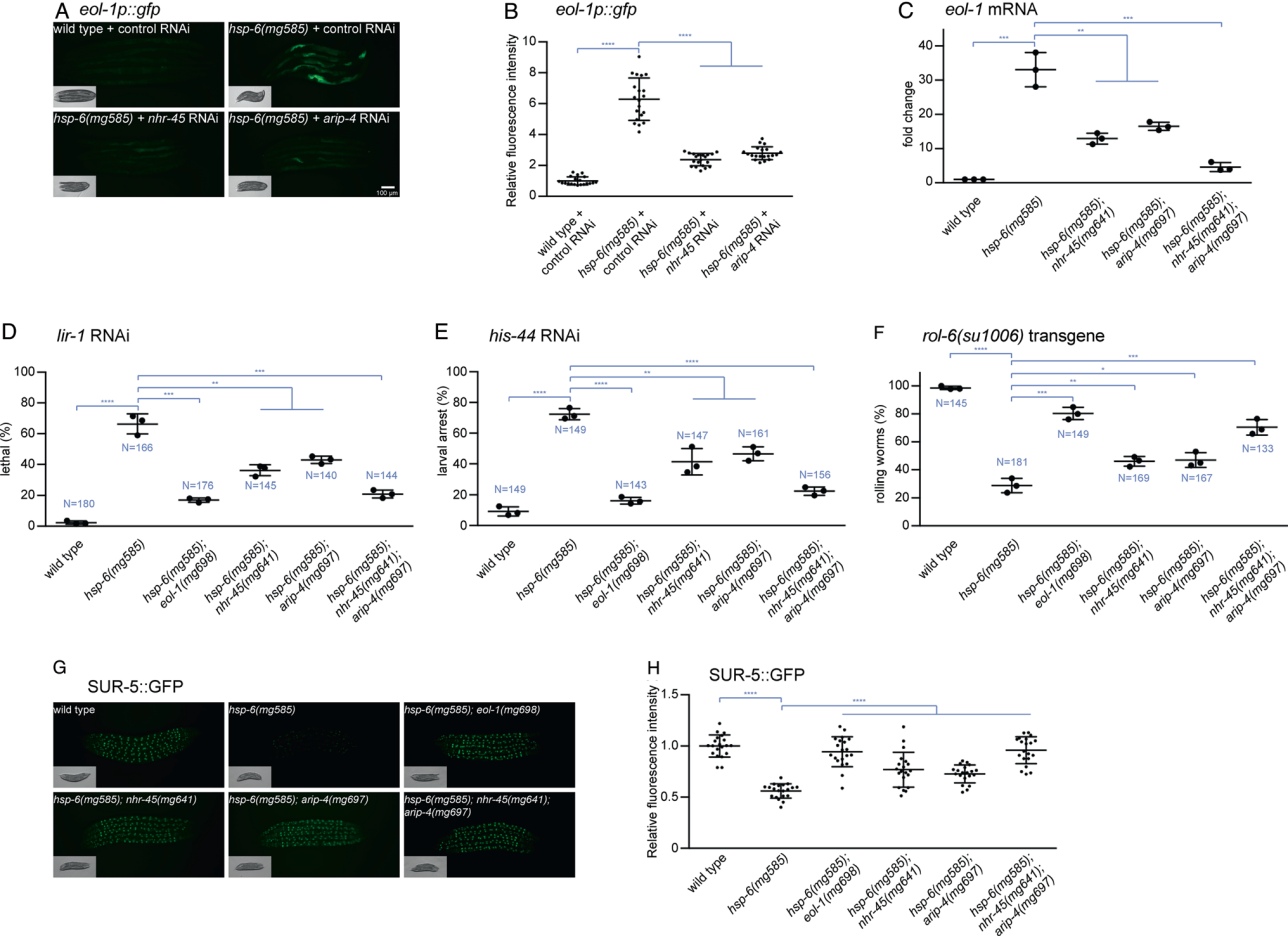
NHR-45 or ARIP-4 modulates mitochondrial dysfunction–induced antiviral RNAi through transcriptional activation of *eol-1* gene. (*A* and *B*) The induction of an *eol-1p::gfp* transcriptional fusion reporter requires *nhr-45* and *arip-4*. The *eol-1p::gfp* is strongly induced by the *hsp-6(mg585)* mitochondrial mutant, and this induction is abrogated by *nhr-45* RNAi and *arip-4* RNAi. Animals were imaged in (*A*), and the fluorescence was quantified in (*B*). **** denotes *P* < 0.0001. (*C*) The *hsp-6(mg585)* mutant causes increased *eol-1* mRNA level in an *nhr-45* and *arip-4*-dependent manner. RT-qPCR assays showed that the mRNA level of *eol-1* was induced by *hsp-6(mg585)* mutant, and this induction was abolished by *nhr-45(mg641)* and *arip-4(mg697)*. ** denotes *P* < 0.01 and *** denotes *P* < 0.001. (*D* and *E*) Enhanced RNAi response to *lir-1* RNAi or *his-44* RNAi in *hsp-6(mg585)* mutant requires *nhr-45(mg641)* and *arip-4(mg697)*. RNAi of *lir-1* or *his-44* causes lethality/arrest on *hsp-6(mg585)* mutant but not wild type. The enhanced RNAi is suppressed by *nhr-45(mg641)* and *arip-4(mg697)*. Results of three replicate experiments are shown. N, total number of animals tested. **** denotes *P* < 0.0001. (*F*) Transgene silencing test with the *rol-6(su1006)* multicopy transgene. The expression of the *rol-6* collagen mutation from the transgene is silenced by the enhanced RNAi in the *hsp-6(mg585)* mutant, and this transgene silencing depends on *nhr-45* and *arip-4* gene activities. Results of three replicate experiments are shown. N, total number of animals tested. **** denotes *P* < 0.0001. (*G* and *H*) Transgene silencing test with SUR-5::GFP transgene. This transgene was ubiquitously expressed in all somatic cells. The expression of the *sur-5::GFP* from the transgene is silenced by enhanced RNAi in the *hsp-6(mg585)* mutant, and this transgene silencing requires *nhr-45* and *arip-4*. Animals were imaged in (*G*), and the fluorescence was quantified in (*H*). **** denotes *P* < 0.0001.

Mitochondrial dysfunction in *C. elegans* also promotes an antiviral RNAi response through activation of the DRH-1/EOL-1 pathway (18). Disruption of mitochondrial homeostasis by the *hsp-6(mg585)* mutant causes release into the cytosol of mRNAs transcribed from the mitochondrial genome, with none of the hallmarks of RNA splicing or other cytoplasmic RNA modifications. These mitochondrial mRNAs are likely to be recognized by DRH-1, the *C. elegans* ortholog of mammalian RIG-I or MDA5, as not transcribed, not spliced, not capped, and not modified in the nucleus (8). Activated DRH-1 then promotes the transcriptional upregulation of the decapping enzyme gene *eol-1*. mRNA-seq comparisons of *hsp-6(mg585)* mutant and *hsp-6(mg585); nhr-45(mg641)* double mutant strains demonstrated that the upregulation of*eol-1* expression requires *nhr-45* (Fig. 2*D*) (23). To verify that the transcriptional upregulation of *eol-1* in the *hsp-6(mg585)* mutant is mediated by *nhr-45*, and to test whether it is also mediated by *arip-4*, a transcriptional fusion reporter *eol-1p::gfp* including the *eol-1* promoter, GFP, and the *eol-1* 3'UTR was employed. The fluorescent signal of *eol-1p::gfp* was almost undetectable in wild-type animals treated with control RNAi and was significantly increased in the *hsp-6(mg585)* mitochondrial mutant with control RNAi (Fig. 3 *A* and *B*). *nhr-45* RNAi or *arip-4* RNAi abolished the activation of *eol-1p::gfp* in the *hsp-6(mg585)* mitochondrial mutant (Fig. 3 *A* and *B*). The *hsp-6(mg585)* mitochondrial mutant enhances RNAi so that transgenes are now recognized as foreign and silenced in mitochondrial mutants (see below). The *eol-1p::gfp* transcriptional fusion reporter is a transgene that is subject to RNAi-based transgene silencing. Therefore, RT-qPCR was performed to accurately evaluate the gene expression of *eol-1* from its normal chromosomal location in these mutants. The mRNA level of *eol-1* in the *hsp-6(mg585)* mutant was increased 30 fold compared with that in wild type (Fig. 3*C*). This induction *eol-1* mRNA in *hsp-6(mg585)* is 2-3× decreased (but still 10x induced relative to wild type) in the *hsp-6(mg585); nhr-45(mg641)* or *hsp-6(mg585); arip-4(mg697)* double mutant, and decreased another 3× in the *hsp-6(mg585); arip-4(mg697); nhr-45(mg641)* triple mutant (Fig. 3*C*). Thus, the transcriptional activation of *eol-1* gene by mitochondrial dysfunction is dependent on *nhr-45* and *arip-4*.

The upregulation of *eol-1* is critical for the enhanced RNAi caused by *hsp-6(mg585)* (23). Enhanced RNAi is an antiviral surveillance and a silencing mechanism that is activated by a variety of mitochondrial mutations (23). We tested whether *nhr-45* and *arip-4* are important for the activation of the antiviral RNAi response upon mitochondrial dysfunction. The *hsp-6(mg585)* mitochondrial mutant shows enhanced RNAi when tested with particular dsRNAs expressed from *Escherichia coli*. For example, a dsRNA targeting the *lir-1* gene causes a far milder phenotype (2% lethal) in wild type than in a variety of enhanced RNAi mutants such as *hsp-6(mg585)* (66% lethal) (Fig. 3*D*). The lethal phenotype of *lir-1* dsRNAs in an enhanced RNAi mutant is due to the siRNA generated from *lir-1* dsRNA targeting the primary transcript from the operon, which includes *lir-1*, *lir-2*, and *lin-26* (*lir-1* is named for **LI**n26-**R**elated) (40). The *eol-1(mg698)* null mutant suppresses the enhanced RNAi of the *hsp-6(mg585)* mutant: the *hsp-6(mg585)*; *eol-1(mg698)* double mutant showed 17% lethality treated with *lir-1* RNAi (Fig. 3*D*) (18). *nhr-45(mg641)* or *arip-4(mg697)* mutations moderately suppressed the enhanced RNAi phenotype in *hsp-6(mg585)* mutant: RNAi of *lir-1* caused 36% and 43% lethality in *hsp-6(mg585)*; *nhr-45(mg641)* and *hsp-6(mg585)*; *arip-4(mg697)* double mutants, respectively, compared with 66% lethality for the *hsp-6(mg585)* single mutant (Fig. 3*D*). Mutations in both *nhr-45* and *arip-4* were synergistic: In the *hsp-6(mg585); nhr-45(mg641); arip-4(mg697)* triple mutant, 21% of animals were lethal after *lir-1* RNAi treatment (Fig. 3*D*).

Another enhanced RNAi dsRNA is *his-44* that encodes a histone 2B gene. Because the DNA sequences of the large histone 2B gene family have a high DNA sequence similarity, RNAi of *his-44* in enhanced RNAi mutants may promiscuously target multiple histone 2B genes to cause lethal arrest. RNAi of *his-44* causes 9% arrest in wild type but 72% arrest in the *hsp-6(mg585)* mutant with enhanced RNAi (Fig. 3*E*). Mutations in *eol-1, nhr-45,* or *arip-4* suppressed the *his-44* enhanced RNAi phenotypes of the *hsp-6* mitochondrial mutant: 16% in the *hsp-6(mg585)*; *eol-1(mg698)* double mutant animals, 41% in *hsp-6(mg585)*; *nhr-45(mg641)* double mutant animals, 46% in *hsp-6(mg585)*; *arip-4(mg697)* double mutant animals, and 22% in *hsp-6(mg585)*; *nhr-45(mg641)*; *arip-4(mg697)* triple mutant animals arrested when treated with *his-44* RNAi (Fig. 3*E*). Thus, the enhanced RNAi response in the *hsp-6(mg585)* mitochondrial mutant requires *nhr-45* and *arip-4* for the induction of *eol-1* to enhance RNAi.

Multicopy *C. elegans* transgenes are silenced in a wide variety of enhanced RNAi mutants, most likely because their assembly during DNA transformation into tandem multicopy extrachromosomal elements marks them as probable foreign genetic elements (41). A chromosomally integrated transgene harboring multiple copies of the *rol-6(su1006)*, a dominant amino acid substitution mutation of a hypodermal collagen, causes 99% of wild-type transgenic animals to have a rolling phenotype (Rol). But if the *hsp-6(mg585)* mitochondrial mutant is crossed into the strain, only 29% of the *hsp-6(mg585)* mutant animals harboring the tandem array transgene is Rol (Fig. 3*F*). An *eol-1* null mutation suppresses this *hsp-6* transgene silencing: The *hsp-6(mg585)*; *eol-1(mg698)* double mutant is 80% Rol (Fig. 3*F*). In the *hsp-6(mg585)*; *nhr-45(mg641)* or *hsp-6(mg585)*; *arip-4(mg697)* double mutant, the *rol-6(su1006)* transgene causes 46% or 47% Rol, respectively, and in the *hsp-6(mg585)*; *nhr-45(mg641)*; *arip-4(mg697)* triple mutant, animals are 70% Rol (Fig. 3*F*). To survey transgene silencing in other tissues, we tested another multicopy tandem array transgene: a SUR-5::GFP fusion gene that ubiquitously expressed in all somatic cells. SUR-5::GFP is strongly expressed in essentially every cell type of wild-type animals but was strongly silenced in the *hsp-6(mg585)* mitochondrial mutant (Fig. 3 *G* and *H*). And this silencing of SUR-5::GFP by the *hsp-6* mitochondrial mutant was strongly suppressed, as shown by bright GFP in the double mutants, by the *eol-1(mg698)*, *nhr-45(mg641),* or *arip-4(mg697)* mutation (Fig. 3 *G* and *H*). Therefore, the transgene silencing and enhanced RNAi phenotypes caused by *hsp-6(mg585)* mutant require *nhr-45* and *arip-4*.

### ARIP-4 Mediates Response to Bacterial Toxins that Attack the Mitochondrion.

**Fig. 4.**
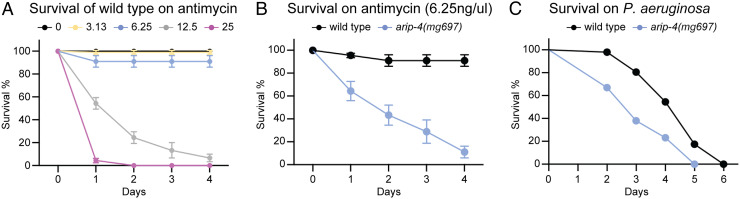
*arip-4* mutant is hypersensitive to antimycin or *P. aeruginosa*. (*A*) Survival of wild-type animals with indicated concentration (μg/ml) of antimycin. (*B*) Survival of wild-type and *arip-4(mg697)* animals with 6.25 μg/ml of antimycin. (*C*) Survival of wild-type and *arip-4(mg697)* animals on *P. aeruginosa*.

The *C. elegans* NHR-45/MDT-15 pathway mediates detoxification of mitochondrial toxins, for example, the *Streptomyces* mitotoxin antimycin or the pathogenic *P. aeruginosa* (23). As the transcriptional coactivator of NHR-45, we tested whether ARIP-4 also acts in this drug and pathogen resistance pathway. The toxicity of antimycin to wild-type animals was established by treating L4 larvae with varying doses of antimycin. Wild-type animals were able to survive as high as 6.25 μg/ml antimycin without significant mortality, but larger doses of 12.5 or 25 μg/ml inhibited growth and survival (Fig. 4*A*). When L4 larvae of wild-type or *arip-4(mg697)* animals were treated with 6.25 μg/ml antimycin, the sensitivity of the *arip-4(mg697)* mutant was dramatically increased relative to wild type (Fig. 4*B*), indicating the critical role of ARIP-4 in the mitochondrial toxin detoxification and response pathway.

*P. aeruginosa* produces chemical toxins that disrupt the mitochondrial membrane potential and virulence factors that inhibit translation (27, 28). The pathogenic response to *P. aeruginosa* was examined by growing *C. elegans* with *P. aeruginosa* as the sole bacterial food. Compared with the wild-type animals, the *arip-4(mg697)* mutants were more susceptible to *P. aeruginosa* (Fig. 4*C*), suggesting that like its binding partner NHR-45, ARIP-4 acts in the pathway for resistance to *P. aeruginosa* mitochondrial toxins. While intestine-specific expression of ARIP-4 is sufficient to allow the induction of *cyp-14A4* responses to mitochondrial dysfunction, we have not tested whether intestine-specific ARIP-4 expression (Fig. 2 *G* and *H*) can rescue the mitochondrial toxin or bacterial pathogen sensitivity of the ARIP-4 mutant (Fig. 4).

### Loss of *arip-4* Extends *C. elegans* Healthy Life Span.

Although the detoxification and immune responses defend the mitochondrion from external insults, such as toxins or pathogens, if the mitochondrial dysfunction is caused by a mutation in a nuclear-encoded or mitochondrially encoded mitochondrial gene, such responses are futile and might actually compromise health and shorten life span (23). Indeed, loss of *nhr-45* improves health status in aged animals and increases life span of the *hsp-6(mg585)* mitochondrial mutant (23). Therefore, we tested whether decoupling mitochondrial response pathways with an *arip-4* mutation might actually increase healthy life span.

**Fig. 5.**
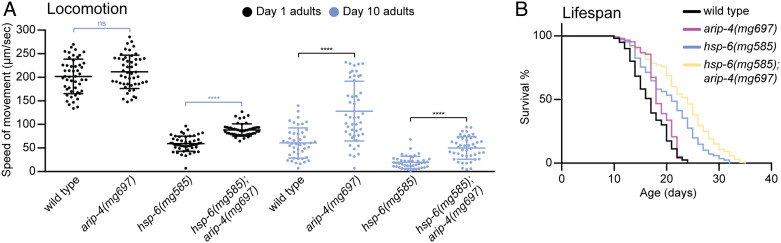
*arip-4* mutant shows an extended healthy life span. (*A*) Locomotion test of day 1 and day 10 adults of wild type and *arip-4(mg697), hsp-6(mg585),* and *hsp-6(mg585); arip-4(mg697)* mutants. **** denotes *P* < 0.0001 and ns denotes *P*>0.05. (*B*) Life span analyses of wild type and *arip-4(mg697), hsp-6(mg585),* and *hsp-6(mg585); arip-4(mg697)* mutants.

The health status of each *C. elegans* genotype was assessed by the relative speed of movement of young (day 1 adults) or aged (day 10 adults) animals. Wild-type animals move at approximately 200 μm/s on day 1, but by day 10, they move 3× more slowly at about 60 μm/s. The movement speed of *hsp-6(mg585)* mitochondrial mutant animals was 59 μm/s at day 1, 30% that of wild type, as would be expected for a mutation that affects mitochondrial production of ATP. At day 10, *hsp-6(mg585)* mitochondrial mutant animals continued to show about 1/3 the motility of wild type with about 20 μm/s (Fig. 5*A*). Compared with wild type, *arip-4(mg697)* did not affect locomotion at day 1 but moved at double the speed (128 μm/s) at day 10 (Fig. 5*A*). An *arip-4* null mutation attenuated the locomotion defects caused by *hsp-6(mg585)* with speed of movement increased 49% on day 1 and 150% on day 10 (Fig. 5*A*).

We suggest that the dramatic increase in health span of *arip-4; hsp-6* mitochondrial mutants is caused by the decoupling of the impotent detoxification and immune responses to mitochondrial dysfunction in the *arip-4(mg697)* mutant to render the animals healthier. For example, cytochrome p450 proteins are heme proteins that require iron, often in limiting supply. The nearly 100× induction of *cyp-14A4* caused by mitochondrial dysfunction may usurp iron from mitochondrial biogenesis, for example. Similarly, secreted antibacterial proteins pass through the endoplasmic reticulum where multiple cysteines are oxidized to disulfides, an energetically expensive modification. Animals bearing mitochondrial mutations that do not mount impotent but stressful detoxification and immune responses because of the second *arip-4* mutation paradoxically fare better. Consistent with this, moderate mitochondrial disruption caused by hypomorphic mutations of essential mitochondrial genes or gene inactivations of nonessential mitochondrial genes extends life span (4, 42). For example, the median survival of *hsp-6(mg585)* (21 d) was substantially increased compared with that of wild type (17 d) (Fig. 5*B*). While the *arip-4(mg697)* single mutant, with a median survival of 18 d, marginally increased the life span compared with wild type, the *hsp-6(mg585); arip-4(mg697)* double mutant lived much longer (24 d) than *hsp-6(mg585)* (Fig. 5*B*), showing that the futile *arip-4*-mediated detoxification response to *hsp-6* mitochondrial dysfunction actually shortens life span.

It was notable that the mortality of *arip-4(mg697)* was extremely low before day 17 (Fig. 5*B*), which was consistent with the improved locomotion at day 10 (Fig. 5*A*). The combined analyses of locomotion and life span indicate that loss of *arip-4*, similar to its binding partner *nhr-45*, extends healthy life span in wild type and in the *hsp-6(mg585)* mutant background, emphasizing the deleterious effects of impotent pathogen defense responses triggered by mutations that cannot be detoxified.

## Discussion

Disturbance of mitochondrial function by toxins, pathogens, or mutations triggers drug detoxification and immune responses through MDT-15 and NHR-45 mediated transcriptional regulation (23). Our genetic screen for mutants that fail to induce detoxification genes in the *hsp-6(mg585)* mitochondrial mutant, which identified the *nhr-45* mutant, also revealed the *arip-4(mg691)*, a nonsense mutation in *arip-4* (Fig. 1 *A* and *B*). *C. elegans arip-4* encodes a conserved Snf2-like DNA-dependent ATPase that is orthologous to human ARIP4 (androgen receptor–interacting protein), which also interacts with a NHR, the androgen receptor. We showed that tagged ARIP-4 and NHR-45 proteins colocalize in the nucleus to coregulate *cyp-*14A4 as well as other genes involved in drug detoxification and antibacterial immunity and that ARIP-4 protein physically interacts with the NHR-45 protein in a similar manner that its human ortholog ARIP4 binds to the NHRs, such as the androgen receptor (35). However, ARIP-4 does not regulate other pathways in mitochondrial homeostasis, such as the gene expression of the mitochondrial chaperone *hsp-6*, which is consistent with our previous analysis of NHR-45 (23). This response allows *C. elegans* to detoxify a mitochondrial toxin antimycin and pathogenic *P. aeruginosa*; loss of this detoxification response in the *mdt-15*, *nhr-45*, or *arip-4* mutants causes sensitivity to antimycin and *P. aeruginosa*. However, this detoxification response becomes deleterious if the mitochondrial defect is a mutation that cannot be neutralized by cytochrome p450 induction or other immune responses.

*nhr-45* and *arip-4* are also important for the activation of the antiviral RNAi response upon mitochondrial dysfunction. The enhanced RNAi and transgene silencing caused by the *hsp-6(mg585)* mitochondrial mutant are moderately suppressed by the *nhr-45(mg641)* or *arip-4(mg697)* single mutant but strongly suppressed by the *nhr-45(mg641); arip-4(mg697)* double mutant (Fig. 3 *D*–*H*). One explanation is that either the *nhr-45(mg641)* or the *arip-4(mg697)* mutant do not completely abolish the upregulation of *eol-1* gene expression in *hsp-6(mg585)* mutant, while the induction of *eol-1* gene is almost absent when both *nhr-45* and *arip-4* were mutated (Fig. 3*C*). This synergy suggests a redundancy of NHR-45 in transcriptional regulation.

NHR-45 is one of the explosion of novel NHRs in the *C. elegans* clade with 284 NHR genes compared with humans with 48 NHR genes (43). NHR genes generally show strong conservation in their Zn-finger DNA-binding domains and less conservation in their ligand-binding domains. A comparison of NHR-45 between divergent parasitic nematodes and more closely Caenorhabditae shows that the NHR-45 ligand-binding domain region (aa 300–500) is also conserved, suggesting a conserved small-molecule ligand. An interesting possibility is that the ligand-binding domain of NHR-45 detects a small molecule such as a particular lipid unique to the mitochondrion that is released by the distressed mitochondria to signal mitochondrial dysfunction. In favor of the model that the NHR-45 ligand-binding domain has an actual ligand, NHR-45 ligand-binding domain detects strong homology with the ligand-binding domains of NHR-86, NHR-102, NHR-142, NHR-178, and NHR-213. These NHR proteins form a clade of related NHR genes that may be regulated by related ligands. A mutation in NHR-86 disrupts the induction of the *irg-4,5, 6* pathogen response genes by 2-N-(3-chloro-4-methylphenyl)quinazoline-2,4-diamine, which may be a ligand of NHR-86 (44, 45). Because such mitochondrial lipid signature molecules may also be synthesized by some species of bacteria, which are ancestrally related to the eukaryotic mitochondrion, NHR-45 may respond to bacterial pathogens as well. Even though NHR-45 has no ortholog in humans, two identified NHR-45 coactivators, ARIP-4/ARIP4 and MDT-15/MED15, are conserved to humans and interact with NHR proteins (37).

## Materials and Methods

### *C. elegans* Strains Used in this Study.

Strains:

**Table:** 

N2	Wild type
HE1006	*rol-6(su1006)* from CGC
MH1046	*Is[sur-5::GFP]* from M. Han lab
GR1831	*mgIs30[lim-6::gfp, col-10::lacZ, rol-6(su1006)]*
GR2249	*hsp-6(mg585) V*
GR2250	*mgIs73[cyp-14A4p::gfp + myo-2p::mcherry] V*
GR2252	*hsp-6(mg585); mgIs73[cyp-14A4p::gfp + myo-2p::mcherry] V*
GR2265	*hsp-6(mg585) V; nhr-45(mg641) X*
GR3309	*hsp-6(mg585); mgIs30*
GR3313	*hsp-6(mg585); Is[sur-5::GFP]*
GR3315	*unc-119(ed3); mgEx864[Peol-1::GFP + cbr-unc-119(+)]*
GR3316	*hsp-6(mg585); unc-119(ed3); mgEx864*
GR3319	*hsp-6(mg585); eol-1(mg698); mgIs30*
GR3320	*hsp-6(mg585); eol-1(mg698); Is[sur-5::GFP]*
GR3331	*rad-26(mg697) IV; mgIs73[cyp-14A4p::gfp + myo-2p::mcherry] V*
GR3332	*rad-26(mg697) IV; hsp-6(mg585); mgIs73[cyp-14A4p::gfp + myo-2p::mcherry] V*
GR3333	*mgTi57[vha-6p::rad-26::gfp]*
GR3334	*hsp-6(mg585) V; mgTi57[vha-6p::rad-26::gfp]*
GR3335	*mgTi57[vha-6p::rad-26::gfp]; mgTi58[vha-6p::nhr-45::mscarlet]*
GR3336	*hsp-6(mg585) V; mgTi57[vha-6p::rad-26::gfp]; mgTi58[vha-6p::nhr-45::mscarlet]*
GR3337	*mgTi57[vha-6p::rad-26::gfp]; mgTi59[vha-6p::nhr-45::flag]*
GR3338	*hsp-6(mg585) V; mgTi57[vha-6p::rad-26::gfp]; mgTi59[vha-6p::nhr-45::flag]*
GR3339	*rad-26(mg697) IV; hsp-6(mg585) V*
GR3340	*rad-26(mg697) IV; hsp-6(mg585); mgIs73[cyp-14A4p::gfp + myo-2p::mcherry] V; mgTi60[vha-6p::rad-26::mscarlet]*
GR3341	*rad-26(mg697) IV; hsp-6(mg585); mgIs73[cyp-14A4p::gfp + myo-2p::mcherry] V; mgTi61[vha-6p::rad-26(K296A)::mscarlet]*

### Generation of Transgenic Animals.

For single-copy transgenes *vha-6p::arip-4::gfp*, *vha-6p::nhr-45::mscarlet*, *vha-6p::nhr-45::flag*, *vha-6::arip-4::mscarlet* and *vha-6::arip-4(K296A)::mscarlet*, the plasmid was injected into *unc-119(ed3)* following the miniMOS protocol (38). For CRISPR of *arip-4(mg697)*, we chose *dpy-10(cn64)* as the co-CRISPR marker (46) and pJW1285 (Addgene) to express both guide RNA (gRNA) and Cas9 enzyme (47).

### Microscopy.

The fluorescent signals of *cyp-14A4p::gfp* transgenic animals were photographed by the Zeiss AX10 Zoom.V16 microscope. The subcellular pattern of *vha-6p::arip-4::gfp* and *vha-6p::nhr-45::mscarlet* was photographed on the Leica TCS SP8 confocal microscope. Photographs were analyzed by the Fiji–ImageJ.

### RNA Isolation and RT-qPCR.

For estimation of the mRNA level of *cyp-14A4*, *ugt-8*, *clec-144*, *cnc-9*, and *hsp-6*, around 200 L4 larvae were handpicked from mixed population and frozen by liquid nitrogen. Total RNA was isolated by TRIzol extraction (Thermo Fisher, 15596026). The cDNA was generated by the ProtoScript^®^ II First Strand cDNA Synthesis Kit (New England Biolabs, E6560L). qPCR was performed toward *cyp-14A4*, *ugt-8*, *clec-144*, *cnc-9,* and *hsp-6* with *act-1* as control by the iQ™ SYBR^®^ Green Supermix (Bio-Rad, 1708880).

### Immunoprecipitation and Western Blot.

Around 20,000 worms of each genotype were synchronized by bleach preparation to L1 larvae, grown to the L4 stage, collected, and frozen by liquid nitrogen. Worm lysates were generated by the TissueLyser with steel beads (Qiagen, 69989) and resuspended in 500 μl lysis buffer (10 mM Tris-HCl, pH 7.5, 150 mM NaCl, 0.5 mM EDTA, 1% Triton X-100, and 1X protease inhibitor cocktail). The lysate was centrifuged at 1,000 g for 10 min at 4°C to remove the pellet debris. And the supernatant was divided into two parts: 30 μl to mix with the NuPAGE™ LDS sample buffer (Thermo Fisher, NP0007) as the input and 450 μl to mix with 20 μl GFP-nAb magnetic agarose (Allele Biotechnology, ABP-NAB-GFPX025), which was pretreated twice by the washing buffer (10 mM Tris-HCl, pH 7.5, 150 mM NaCl, and 0.5 mM EDTA). The mixture was rotated for 2 h at 4°C to allow the binding. The beads were washed three times with the washing buffer and resuspended in 50 μl 1X NuPAGE™ LDS sample buffer. The input and the beads were heated at 70°C for 10 min. Samples were loaded onto the NuPAGE™ 4 to 12% Bis-Tris protein gels (Thermo Fisher, NP0323BOX) and run with the NuPAGE™ MES SDS running buffer (Thermo Fisher, NP0002). After semidry transfer, the PVDF membrane (Millipore, IPVH00010) was blocked with 5% nonfat milk and probed with anti-GFP (Fisher Scientific, NC9777966) or anti-FLAG (Sigma, F1804) primary antibody and goat anti-mouse IgG HRP secondary antibody (Thermo Fisher, 31430). The membrane was developed with the SuperSignal™ West Femto Maximum Sensitivity Substrate (Thermo Fisher, 34096) and visualized by the Amersham Hyperfilm (GE Healthcare, 28906845).

### Antimycin Treatment.

For each experiment, 30 L4 larvae were treated with different concentrations of antimycin A (Sigma, A8674), and each experiment was repeated three times. To determine the sensitivity of wild-type animals, a twofold serial dilution was performed to antimycin starting from 25 μg/ml. For comparing the sensitivity of wild-type and *arip-4(mg697)* animals, 6.25 μg/ml antimycin was used. Survival was examined on a daily basis, and the survival curve was generated by GraphPad Prism.

### *P. aeruginosa* Pathogenic Assay.

*P. aeruginosa* PA14 was grown at 37°C overnight, seeded on the NGM plates, and incubated at 37°C for another night. One hundred L4 larvae were picked onto *P. aeruginosa* lawn and grown at 25°C. Survival was examined on a daily basis, and the survival curve was generated by GraphPad Prism.

### Examination of Locomotion.

Fifty L4 larvae were picked and grown at 20°C for 10 d. On day 1 and day 10, worms were picked onto bacteria-free NGM plates and photographed directly by the Zeiss AX10 Zoom.V16 microscope. The movement of worms was recorded by continuing picturing every 0.5 s for 30 s in total. The speed of movement was analyzed and calculated by the AxioVision (Zeiss).

### Life Span Analysis.

Animals were synchronized by egg laying and grown until the L4 stage as day 0. Adults were separated from their progenies by manually transferring to new plates. Survival was examined on a daily basis, and the survival curve was generated by GraphPad Prism.

## Data Availability

All study data are included in the article.

## References

[1] C. Lopez-Otin, M. A. Blasco, L. Partridge, M. Serrano, G. Kroemer, The hallmarks of aging. Cell **153**, 1194–1217 (2013). 23746838 10.1016/j.cell.2013.05.039 PMC3836174

[2] D. E. Shore, G. Ruvkun, A cytoprotective perspective on longevity regulation. Trends Cell Biol. **23**, 409–420 (2013). 23726168 10.1016/j.tcb.2013.04.007 PMC4057428

[3] C. E. Riera, A. Dillin, Emerging role of sensory perception in aging and metabolism. Trends Endocrinol. Metab. **27**, 294–303 (2016). 27067041 10.1016/j.tem.2016.03.007

[4] S. S. Lee , A systematic RNAi screen identifies a critical role for mitochondria in C. elegans longevity. Nat. Genet. **33**, 40–48 (2003). 12447374 10.1038/ng1056

[5] A. J. Roger, S. A. Munoz-Gomez, R. Kamikawa, The origin and diversification of mitochondria. Curr. Biol. **27**, R1177–R1192 (2017). 29112874 10.1016/j.cub.2017.09.015

[6] A. Nerlich , Pneumolysin induced mitochondrial dysfunction leads to release of mitochondrial DNA. Sci. Rep. **8**, 182 (2018). 29317705 10.1038/s41598-017-18468-7 PMC5760655

[7] S. Gupta, G. V. Prasad, A. Mukhopadhaya, Vibrio cholerae porin OmpU induces caspase-independent programmed cell death upon translocation to the host cell mitochondria. J. Biol. Chem. **290**, 31051–31068 (2015). 26559970 10.1074/jbc.M115.670182 PMC4692230

[8] J. Rehwinkel, M. U. Gack, RIG-Ilike receptors: Their regulation and roles in RNA sensing. Nat. Rev. Immunol. **20**, 537–551 (2020). 32203325 10.1038/s41577-020-0288-3 PMC7094958

[9] M. Tigano, D. C. Vargas, S. Tremblay-Belzile, Y. Fu, A. Sfeir, Nuclear sensing of breaks in mitochondrial DNA enhances immune surveillance. Nature **591**, 477–481 (2021). 33627873 10.1038/s41586-021-03269-w

[10] A. P. West, G. S. Shadel, Mitochondrial DNA in innate immune responses and inflammatory pathology. Nat. Rev. Immunol. **17**, 363–375 (2017). 28393922 10.1038/nri.2017.21 PMC7289178

[11] Y. Tabach , Identification of small RNA pathway genes using patterns of phylogenetic conservation and divergence. Nature **493**, 694–698 (2013). 23364702 10.1038/nature11779 PMC3762460

[12] R. Lu , Animal virus replication and RNAi-mediated antiviral silencing in Caenorhabditis elegans. Nature **436**, 1040–1043 (2005). 16107851 10.1038/nature03870 PMC1388260

[13] C. Wilkins , RNA interference is an antiviral defence mechanism in Caenorhabditis elegans. Nature **436**, 1044–1047 (2005). 16107852 10.1038/nature03957

[14] D. B. Gammon , The antiviral RNA interference response provides resistance to lethal arbovirus infection and vertical transmission in caenorhabditis elegans. Curr. Biol. **27**, 795–806 (2017). 28262484 10.1016/j.cub.2017.02.004 PMC5446062

[15] A. Ashe , A deletion polymorphism in the Caenorhabditis elegans RIG-I homolog disables viral RNA dicing and antiviral immunity. Elife **2**, e00994 (2013). 24137537 10.7554/eLife.00994 PMC3793227

[16] A. Dhir , Mitochondrial double-stranded RNA triggers antiviral signalling in humans. Nature **560**, 238–242 (2018). 30046113 10.1038/s41586-018-0363-0 PMC6570621

[17] A. Pajak , Defects of mitochondrial RNA turnover lead to the accumulation of double-stranded RNA in vivo. PLoS Genet. **15**, e1008240 (2019). 31365523 10.1371/journal.pgen.1008240 PMC6668790

[18] K. Mao, P. Breen, G. Ruvkun, Mitochondrial dysfunction induces RNA interference in C. elegans through a pathway homologous to the mammalian RIG-I antiviral response. PLoS Biol. **18**, e3000996 (2020). 33264285 10.1371/journal.pbio.3000996 PMC7735679

[19] S. E. J. Fischer, G. Ruvkun, Caenorhabditis elegans ADAR editing and the ERI-6/7/MOV10 RNAi pathway silence endogenous viral elements and LTR retrotransposons. Proc. Natl. Acad. Sci. U.S.A. **117**, 5987–5996 (2020). 32123111 10.1073/pnas.1919028117 PMC7084138

[20] T. Shpilka, C. M. Haynes, The mitochondrial UPR: Mechanisms, physiological functions and implications in ageing. Nat. Rev. Mol. Cell Biol. **19**, 109–120 (2018). 29165426 10.1038/nrm.2017.110

[21] J. A. Melo, G. Ruvkun, Inactivation of conserved C. elegans genes engages pathogen- and xenobiotic-associated defenses. Cell **149**, 452–466 (2012). 22500807 10.1016/j.cell.2012.02.050 PMC3613046

[22] Y. Liu, B. S. Samuel, P. C. Breen, G. Ruvkun, Caenorhabditis elegans pathways that surveil and defend mitochondria. Nature **508**, 406–410 (2014). 24695221 10.1038/nature13204 PMC4102179

[23] K. Mao , Mitochondrial dysfunction in C. elegans activates mitochondrial relocalization and nuclear hormone receptor-dependent detoxification genes. Cell Metab. **29**, 1182–1191.e1184 (2019). 30799287 10.1016/j.cmet.2019.01.022 PMC6506380

[24] P. A. Frankino, E. A. Moehle, A. Dillin, Evolutionary comeuppance: Mitochondrial stress awakens the remnants of ancient bacterial warfare. Cell Metab. **29**, 1015–1017 (2019). 31067444 10.1016/j.cmet.2019.04.007

[25] J. H. Thomas, Analysis of homologous gene clusters in Caenorhabditis elegans reveals striking regional cluster domains. Genetics **172**, 127–143 (2006). 16291650 10.1534/genetics.104.040030 PMC1456141

[26] O. Thompson , The million mutation project: A new approach to genetics in Caenorhabditis elegans. Genome Res. **23**, 1749–1762 (2013). 23800452 10.1101/gr.157651.113 PMC3787271

[27] N. V. Kirienko, F. M. Ausubel, G. Ruvkun, Mitophagy confers resistance to siderophore-mediated killing by Pseudomonas aeruginosa. Proc. Natl. Acad. Sci. U.S.A. **112**, 1821–1826 (2015). 25624506 10.1073/pnas.1424954112 PMC4330731

[28] D. L. McEwan, N. V. Kirienko, F. M. Ausubel, Host translational inhibition by Pseudomonas aeruginosa exotoxin a triggers an immune response in Caenorhabditis elegans. Cell Host Microbe **11**, 364–374 (2012). 22520464 10.1016/j.chom.2012.02.007 PMC3334877

[29] S. S. Patel, I. Donmez, Mechanisms of helicases. J. Biol. Chem. **281**, 18265–18268 (2006). 16670085 10.1074/jbc.R600008200

[30] P. Umate, N. Tuteja, R. Tuteja, Genome-wide comprehensive analysis of human helicases. Commun. Integr. Biol. **4**, 118–137 (2011). 21509200 10.4161/cib.4.1.13844 PMC3073292

[31] S. Li, Transcription coupled nucleotide excision repair in the yeast Saccharomyces cerevisiae: The ambiguous role of Rad26. DNA Repair (Amst) **36**, 43–48 (2015). 26429063 10.1016/j.dnarep.2015.09.006

[32] G. J. Narlikar, R. Sundaramoorthy, T. Owen-Hughes, Mechanisms and functions of ATP-dependent chromatin-remodeling enzymes. Cell **154**, 490–503 (2013). 23911317 10.1016/j.cell.2013.07.011 PMC3781322

[33] A. Domanskyi, K. T. Virtanen, J. J. Palvimo, O. A. Janne, Biochemical characterization of androgen receptor-interacting protein 4. Biochem. J. **393**, 789–795 (2006). 16212558 10.1042/BJ20050823 PMC1360732

[34] H. Ogawa, T. Komatsu, Y. Hiraoka, K. Morohashi, Transcriptional suppression by transient recruitment of ARIP4 to sumoylated nuclear receptor Ad4BP/SF-1. Mol. Biol. Cell **20**, 4235–4245 (2009). 19692572 10.1091/mbc.E08-12-1247 PMC2754937

[35] N. Rouleau , Novel ATPase of SNF2-like protein family interacts with androgen receptor and modulates androgen-dependent transcription. Mol. Biol. Cell. **13**, 2106–2119 (2002). 12058073 10.1091/mbc.01-10-0484. PMC117628

[36] F. P. Zhang , An adenosine triphosphatase of the sucrose nonfermenting 2 family, androgen receptor-interacting protein 4, is essential for mouse embryonic development and cell proliferation. Mol. Endocrinol. **21**, 1430–1442 (2007). 17374848 10.1210/me.2007-0052

[37] J. D. McGhee, “C. elegans intestine”. in WormBook (WormBook, 2007), pp.1–36, 10.1895/wormbook.1.133.1. PMC4780959 18050495

[38] C. Frokjaer-Jensen , Random and targeted transgene insertion in Caenorhabditis elegans using a modified Mos1 transposon. Nat. Methods **11**, 529–534 (2014). 24820376 10.1038/nmeth.2889 PMC4126194

[39] O. Cohen-Fix, P. Askjaer, Cell biology of the Caenorhabditis elegans nucleus. Genetics **205**, 25–59 (2017). 28049702 10.1534/genetics.116.197160 PMC5216270

[40] D. M. Pavelec, J. Lachowiec, T. F. Duchaine, H. E. Smith, S. Kennedy, Requirement for the ERI/DICER complex in endogenous RNA interference and sperm development in Caenorhabditis elegans. Genetics **183**, 1283–1295 (2009). 19797044 10.1534/genetics.109.108134 PMC2787421

[41] S. E. Fischer , Multiple small RNA pathways regulate the silencing of repeated and foreign genes in C. elegans. Genes Dev. **27**, 2678–2695 (2013). 24352423 10.1101/gad.233254.113 PMC3877757

[42] A. Dillin , Rates of behavior and aging specified by mitochondrial function during development. Science **298**, 2398–2401 (2002). 12471266 10.1126/science.1077780

[43] M. Robinson-Rechavi, C. V. Maina, C. R. Gissendanner, V. Laudet, A. Sluder, Explosive lineage-specific expansion of the orphan nuclear receptor HNF4 in nematodes. J. Mol. Evol. **60**, 577–586 (2005). 15983867 10.1007/s00239-004-0175-8

[44] N. D. Peterson , The nuclear hormone receptor NHR-86 controls anti-pathogen responses in C. elegans. PLoS Genet. **15**, e1007935 (2019). 30668573 10.1371/journal.pgen.1007935 PMC6358101

[45] H. E. Arda , Functional modularity of nuclear hormone receptors in a Caenorhabditis elegans metabolic gene regulatory network. Mol. Syst. Biol. **6**, 367 (2010). 20461074 10.1038/msb.2010.23 PMC2890327

[46] J. A. Arribere , Efficient marker-free recovery of custom genetic modifications with CRISPR/Cas9 in Caenorhabditis elegans. Genetics **198**, 837–846 (2014). 25161212 10.1534/genetics.114.169730 PMC4224173

[47] J. D. Ward, Rapid and precise engineering of the Caenorhabditis elegans genome with lethal mutation co-conversion and inactivation of NHEJ repair. Genetics **199**, 363–377 (2015). 25491644 10.1534/genetics.114.172361 PMC4317648

